# Next-generation sequencing yields the complete chloroplast genome of *Abies kawakamii*

**DOI:** 10.1080/23802359.2018.1535856

**Published:** 2018-11-25

**Authors:** Yi-Zhen Shao, Yun Chen, Xiao-Fang Lu, Yong-Zhong Ye, Zhi-Liang Yuan

**Affiliations:** College of Life Sciences, Henan Agriculture University, Zhengzhou, China

**Keywords:** *Abies kawakamii*, chloroplast genome, threatened, phylogenetic

## Abstract

*Abies kawakamii* is endemic to the island of Taiwan and has been listed as a threatened species in the Red List. In present study, we reported the complete chloroplast genome of *A. kawakamii*. The chloroplast genome is 121,290 bp in size. It was composed of 114 genes and they were 68 peptide-encoding genes, 35 tRNA genes, four rRNA genes, six open reading frames and one pseudogene. Loss of ndh genes was identified in the genome of *A*. *kawakamii*. Inverted repeat sequences include trnS–psaM–ycf12–trnG and trnG–ycf12–psaM–trnS were recognized in 52-kb inversion points. The phylogenetic analysis confirms that the *Abies* species are strongly supported as monophyletic. The complete plastome of *A*. *kawakamii* will provide potential genetic resources for further conservation and management strategies.

*Abies kawakamii* (Hay.) Ito is endemic to the island of Taiwan and has been listed as a threatened species in the Red List (IUCN 2018). It is one of the southernmost firs (together with *A. fansipanensis*, native to Vietnam, and *A. guatemalensis*, from Mexico and Guatemala) (Liu 1971; Farjon 2001; Xiang et al. 2015). The Taiwan fir adapted to a cold, humid environment at high elevations and typically occurs in sheltered to windswept sites (Huang 2002). In the present study, we assembled and characterized the complete plastome of *A*. *kawakamii*. It will be fundamental to formulate conservation and management strategies for this threatened species.

We collected the plant material from the Hehuan Mountain of Taiwan. The voucher specimen (Chen C.-J., No. 9471) was deposited at the herbarium of Institute of Botany, CAS (PE). Complete chloroplast (cp) genome of *Abies kawakamii* was sequenced by HiSeq4000 of Illumina. Totally 10.3 million high-quality clean reads (150 bp PE read length) were obtained. In total, ca. 10.1 million high-quality clean reads (150 bp PE read length) were generated with adaptors trimmed. The CLC de novo assembler (CLC Bio, Aarhus, Denmark), BLAST, GeSeq (Tillich et al. 2017), and tRNAscan-SE v1.3.1 (Schattner et al. 2005) were used to align, assemble, and annotate the plastome.

The complete chloroplast genome consists of 121,290 bp for *Abies kawakamii* (GenBank: MH706726). The circle genome was comprised of a large single copy region (LSC with 65,646 bp), a small single copy region (SSC with 55,116 bp), and two inverted repeat regions (IR with 264 bp). The overall GC content of the *A*. *kawakamii* cp genome was 38.3%. It was composed of 114 genes and they were 68 peptide-encoding genes, 35 tRNA genes, four rRNA genes, six open reading frames and one pseudogene. All ndh genes have been lost in the genome of *A*. *kawakamii*. Short inverted repeat sequences were detected in 52-kb inversion points of the cp genome, which consist of trnS–psaM–ycf12–trnG and trnG–ycf12–psaM–trnS (1183 bp). Interestingly, such inverted repeats had been reported in several members of the genus *Abies* (*A. koreana* and *A*. *ziyuanensis*) (Yi et al. 2015; Shao et al. 2018).

To infer the phylogenetic position of *Abies kawakamii*, nine chloroplast genomes were selected in Pinaceae with *Ginkgo biloba* (Ginkgoaceae) as the outgroup. These sequences were fully aligned with MAFFT v7.3 (Suita, Osaka, Japan) (Katoh and Standley 2013), and the maximum likelihood (ML) inference was performed using GTRþIþC model with RAxML v.8.2.1 (Karlsruhe, Germany) (Stamatakis 2014) on the CIPRES cluster service (Miller et al. 2010). The two *Abies* species (*A. kawakamii* and *A. beshanenzuensis*) were found to be a monophyletic group (Figure 1).

**Figure 1.**
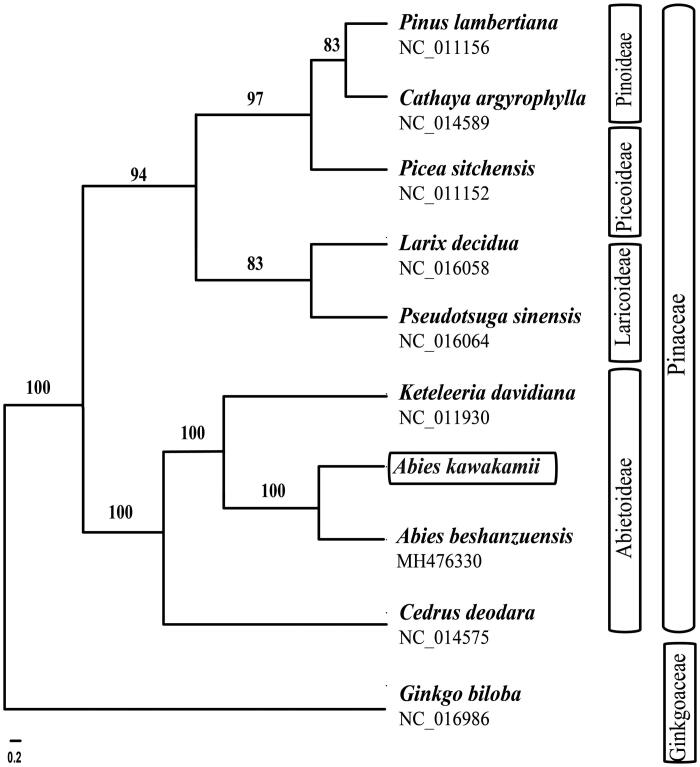
The best maximum-likelihood (ML) phylogram inferred from ten chloroplast genomes in Pinaceae and Ginkgoaceae (bootstrap value are indicated on the branches).
